# Separation of Risks and Benefits of Seafood Intake

**DOI:** 10.1289/ehp.9738

**Published:** 2006-12-14

**Authors:** Esben Budtz-Jørgensen, Philippe Grandjean, Pal Weihe

**Affiliations:** 1 Department of Biostatistics, Institute of Public Health, University of Copenhagen, Copenhagen, Denmark; 2 Institute of Public Health, University of Southern Denmark, Odense, Denmark; 3 Department of Environmental Health, Harvard School of Public Health, Boston, Massachusetts, USA; 4 Faroese Hospital System, Tórshavn, Faroe Islands

**Keywords:** confounding factors, exposure assessment, food contamination, methylmercury compounds, prenatal exposure–delayed effects, seafood

## Abstract

**Background:**

Fish and seafood provide important nutrients but may also contain toxic contaminants, such as methylmercury. Advisories against pollutants may therefore conflict with dietary recommendations. In resolving this conundrum, most epidemiologic studies provide little guidance because they address either nutrient benefits or mercury toxicity, not both.

**Objectives:**

Impact on the same health outcomes by two exposures originating from the same food source provides a classical example of confounding. To explore the extent of this bias, we applied structural equation modeling to data from a prospective study of developmental methylmercury neurotoxicity in the Faroe Islands.

**Results:**

Adjustment for the benefits conferred by maternal fish intake during pregnancy resulted in an increased effect of the prenatal methylmercury exposure, as compared with the unadjusted results. The dietary questionnaire response is likely to be an imprecise proxy for the transfer of seafood nutrients to the fetus, and this imprecision may bias the confounder-adjusted mercury effect estimate. We explored the magnitude of this bias in sensitivity analysis assuming a range of error variances. At realistic imprecision levels, mercury-associated deficits increased by up to 2-fold when compared with the unadjusted effects.

**Conclusions:**

These results suggest that uncontrolled confounding from a beneficial parameter, and imprecision of this confounder, may cause substantial underestimation of the effects of a toxic exposure. The adverse effects of methylmercury exposure from fish and seafood are therefore likely to be underestimated by unadjusted results from observational studies, and the extent of this bias will be study dependent.

Fish and seafood are of environmental health interest because of the biomagnification of persistent toxicants in freshwater and marine food chains, thereby providing an important pathway for human exposures. At the same time, fish may also constitute an important source of energy, protein, and essential micronutrients, thus providing health benefits. For these reasons, risk managers and health communicators must reconcile advisories against pollutants and recommendations on nutrient intakes (Gochfeld and Burger 2005; Levenson and Axelrad 2006; Smith and Sahyoun 2005). Thus, an international expert committee (Joint Expert Committee on Food Additives 2003)

Recommended that nutritional benefits be weighed against the possibility of harm when limits on the methylmercury concentrations in fish or on fish consumption are being considered.

Less attention has been paid to this challenge in regard to the underlying epidemiologic documentation, where exposures to contaminants and beneficial nutrients may be highly correlated in frequent fish-eaters (Sakamoto et al. 2004). On one hand, methylmercury exposure may adversely affect the neurobehavioral development in children (Grandjean et al. 2005b), and mercury contamination is now the main reason for fishing advisories in the United States [U.S. Environmental Protection Agency (EPA) 2004]. On the other hand, nutrients in fish and seafood, especially long-chained n-3 polyunsaturated fatty acids, may affect the same type of outcomes, although in the opposite direction (Daniels et al. 2004; Willatts and Forsyth 2000). Unfortunately, the great majority of cohort studies in this field has focused either on contaminant risks or on nutrient benefits.

This situation appears to constitute a classical example of confounding, where the factors that affect the same outcome are associated—in this case because they derive from the same type of food items. If confounding is not addressed in the epidemiologic study design or the data analysis, the effect of both the contaminant exposure and the nutrient intake will be underestimated.

Only a few studies have aimed at examining the effects of both nutrient and contaminant intakes at the same time as predictors of developmental outcomes. One small study of neurodevelopment in infants suggested that maternal mercury exposure and fish intake had opposite effects on a visually mediated neurobehavioral test (Oken et al. 2005). In another study, Daniels et al. (2004) saw a beneficial association with fish intake and no clear effect of low mercury concentrations in umbilical cord tissue. In a small Faroese birth cohort, Steuerwald et al. (2000) found that prenatal methylmercury exposure adversely affected neonatal neurologic function, but selenium and n-3 fatty acid status did not affect this outcome. All of these results were probably affected by the imprecision of the mercury exposure parameters, which may bias the findings toward the null hypothesis and exaggerate the effects of confounding (Budtz-Jørgensen et al. 2003).

To examine the possibility of segregating benefits and risks, we have analyzed data from a prospective birth cohort study carried out in the Faroe Islands to assess the developmental neurotoxicity of methylmercury from seafood (Debes et al. 2006; Grandjean et al. 1992, 1997).

## Methods

### Cohort formation and clinical follow-up

A birth cohort of 1,022 subjects was formed from consecutive births between 1 March 1986 and the end of 1987 at the three Faroese hospitals (Grandjean et al. 1992). In connection with each birth, we collected umbilical cord blood and maternal hair for mercury analysis. A questionnaire was administered by the midwife to obtain basic information on the general course of the pregnancy and nutritional habits, including the average number of fish dinners per week during pregnancy. Follow-up of this cohort included an extensive neurobehavioral examination at 7 years of age (Grandjean et al. 1997) and 14 years of age (Debes et al. 2006), at which neurobehavioral tests were administered by clinical professionals. About 90% of the cohort children participated in the follow-up. Parents of the children gave written informed consent, and the study was carried out in accordance with the Helsinki convention and related regulations with the approval of the ethical review committee for the Faroe Islands and the institutional review board in the United States.

### Statistical analysis

We carried out a structural equation model analysis as previously described (Budtz-Jørgensen et al. 2002). In these models, observed variables are considered manifestations of a limited number of causally related latent variables. This approach is useful for analyzing multidimensional epidemiologic data. Although multiple regression analysis provides an effect estimate for each pair of exposure and outcome variables, it is prone to multiple testing problems and chance findings. In contrast, the structural equation model pools information from variables measuring the same underlying quantity to obtain a stronger and more parsimonious analysis of the dose–response relationship. Furthermore, this model class allows for measurement error in observed variables. In the current application, log-transformed mercury concentrations in cord blood and maternal hair were considered indicators of the unobserved true exposure level. Using a factor analysis approach, each marker of mercury exposure (*M-Hg*) is assumed to depend linearly on a latent true exposure (*Hg*) and a random measurement error (ɛ*_m_*):


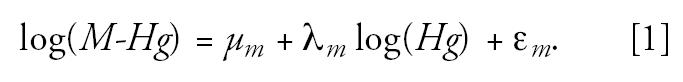


**Figure 1:**
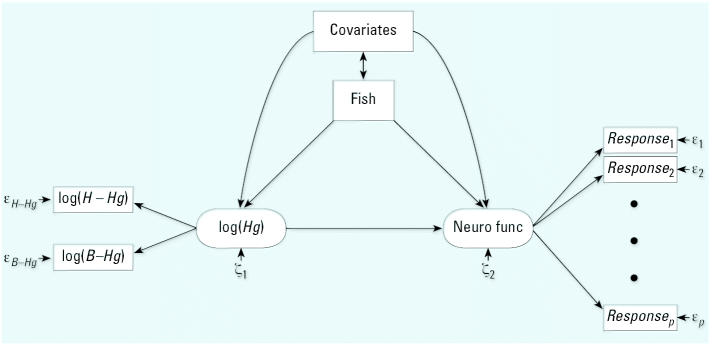
Path diagram for a structural equation model that links mercury exposure to adverse effects, while taking into account confounders, including fish intake. The exposure (*Hg*) is modeled as a latent parameter based on available exposure indicators, and the latent effect parameter [neurologic function (Neuro func)] is likewise based on clinical test outcomes. Each of the exposure indicators and clinical outcomes is associated with imprecision (ɛ).

Based on *a priori* neurobehavioral knowledge and supported by exploratory factor analysis, the outcome variables were grouped into major nervous system functions, as previously described (Budtz-Jørgensen et al. 2002; Debes et al. 2006). Using equations similar to Equation 1, test scores belonging to the same function group were assumed to reflect a common latent outcome function. For each group of neurobehavioral tests, we estimated the effect of mercury by regression of the latent exposure on the latent outcome (Figure 1). The mercury effect was expressed in terms of the change in the latent response variable (in percent of its SD) associated with a doubling in the latent mercury exposure, as has been done previously for outcomes on different scales (Grandjean et al. 1999). The statistical significance of the mercury effect was evaluated using likelihood ratio testing. Children with incomplete information—mainly due to missing maternal Raven score (Budtz-Jørgensen et al. 2002; Debes et al. 2006)—were included by a missing data analysis based on the maximum likelihood principle (Little and Rubin 2002).

Confounders included a series of covariates as previously described (Budtz-Jørgensen et al. 2007; Grandjean et al. 1997); polychlorinated biphenyl (PCB) exposure was not considered a covariate because it had a limited impact on the mercury effects and was unavailable for the majority of the cohort members (Debes et al. 2006; Grandjean et al. 2001). As an extension of the previous analyses, the log-transformed number of maternal fish dinners during pregnancy was included as a covariate that was allowed to affect both prenatal exposure and outcomes. However, this questionnaire parameter provides only an error-prone reflection of the nutrient intake. The analysis described above does not account for the confounder imprecision and may therefore not properly separate the effects of mercury and nutrients from fish. Thus, ignoring the confounder imprecision can lead to biased estimates not only for the confounder effect but also for the effect of the exposure. Consider a regression model with exposure *X* and true confounder *Z*, that is,


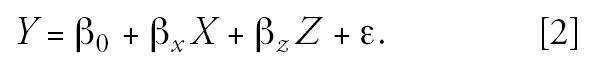


Instead of the true confounder *Z*, we observe an error-prone proxy variable *V*. Here we assume that *V* has an additive error, that is, *V = Z + U*, where *U* is a nondifferential measurement error. If this error is ignored and *Z* is naively replaced by *V* in the regression analysis, then the regression coefficient for the exposure estimate is biased. As the number of observations increase, the least-squares estimator will not converge to the true effect β*_x_*, but to


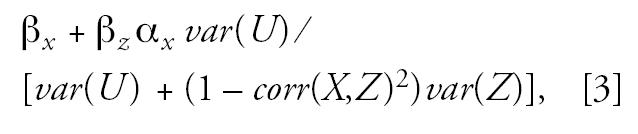


where α*_x_* is the coefficient of *X* in the regression of *Z* on *X*, and *corr(X,Z)* is the correlation between *X* and *Z*. In the special case where *X* is mercury exposure and *Z* is nutrient intake from fish, the effect of *Z* on *Y*(β*_z_*) is positive, and so is the slope in the relation between mercury exposure and nutrients intake α_x_. Therefore, the bias term is positive, and the adverse effect of mercury is underestimated. As can be seen from Equation 3, this bias will be stronger for an increased imprecision *var(U),* a stronger association between exposure and confounder [α*_x_, corr(X,Y)*], and a stronger effect of the confounder β*_z_*.

**Figure 2:**
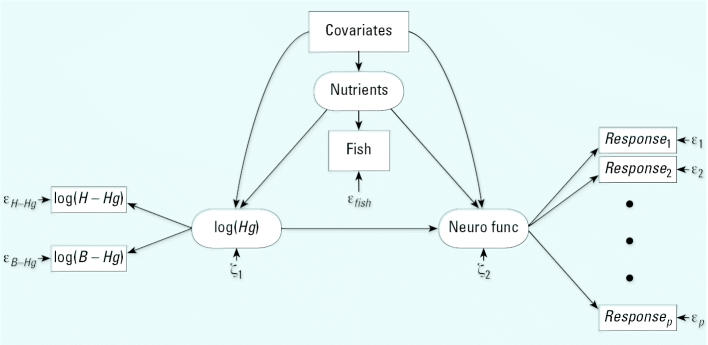
Path diagram for a structural equation model that links mercury exposure to adverse effects, while taking into account confounders, including nutrient supply based on fish intake. The exposure (*Hg*) is modeled as a latent parameter based on available exposure indicators, and the latent effect parameter [neurologic function (Neuro func)] is likewise based on clinical test outcomes. Each of the exposure indicators and clinical outcomes is associated with imprecision (ɛ).

In the absence of validation data, we carried out sensitivity analyses to assess the dependence of the results on the imprecision of fish intake as a confounder. In a separate structural equation model, the fish variable was assumed to be a sum of true nutrient intake and a random measurement error (Figure 2). Four different imprecision levels were then chosen to cover a realistic range and expressed in terms of the reliability ratio, namely, the ratio between the variance of the true variable and the total variance observed {i.e., *[var(V)*−*var(U)]/var(V)*}. The percentage of the total variation caused by measurement error is 1 – reliability ratio.

## Results

Half of the mothers had fish for dinner at least three times per week during pregnancy, and only 2% ate fish for dinner less than once per week. The mercury concentrations varied considerably, covering a span of almost 1,000-fold. Although mercury exposure in this population mainly originated from ingestion of whale meat (Grandjean et al. 1992), the log-transformed frequency of fish dinners correlated significantly with log-transformed mercury concentrations in cord blood (*r* = 0.25, *p* < 0.0001) and maternal hair (*r* = 0.26, *p* < 0.0001). Because intake of seafood nutrients essential for nervous system development would be associated with the dietary intake level, this parameter was therefore treated as a confounder in regard to neurobehavioral development outcomes in this cohort.

After adjustment for fish intake in a structural equation model (Figure 1), previously reported mercury regression coefficients (Budtz-Jørgensen et al. 2002; Debes et al. 2006; Grandjean et al. 1997) changed toward a larger mercury effect. At the same time, the *p*-values for the mercury effect decreased (Table 1). Fish intake had a beneficial effect on all seven outcome functions considered. However, this effect was statistically significant only for the motor function outcomes, both at 7 and 14 years of age, and spatial functioning at 14 years. For these outcomes, the effect of increasing the weekly number of fish dinners from 0 to 1 (or from 1 to 3) led to improved test performance between 17% and 25% of the SD of the outcome. If included in the model without mercury exposure, the beneficial effects of fish intake were weaker and less significant; one outcome parameter (verbal at 7 years of age) showed a fish effect in the opposite direction, thus indicating an adverse effect.

The estimated regression coefficients may be biased because of imprecision of the fish variable. The extent of this bias was explored by including nutrient intake as a latent confounder variable, which—together with a random error—affected the questionnaire response on fish dinners (Figure 2). Because the degree of imprecision of the proxy variable is unknown, a range of imprecision levels were entered to explore the effect on the mercury regression coefficients. When the imprecision of the fish variable increased, the adverse mercury effects became stronger and more significant. In accordance with Equation 3, the results were most sensitive for outcomes with a strong fish effect (i.e., motor and spatial functions) (Table 2). At a reliability ratio < 43%, the mercury effects on motor function doubled, compared with the unadjusted results. For the spatial function, the mercury coefficient became negative, indicating an adverse effect, but even for the highest level of imprecision considered, the *p*-value remained > 5%. Similarly, the other outcomes showed stronger mercury effects when the imprecision in the fish variable was increased. However, because of the weaker impact of the fish parameter, the increases in the mercury effect were less dramatic. Thus, even when reliability ratio was only 27% percent, the mercury coefficients increased by ≤ 5% of the outcome SD, and the *p*-values remained stable.

## Discussion

These results support the notion that confounding may be of importance when exposure to a toxicant occurs from a food source that is also associated with essential or otherwise beneficial nutrients. Such confounding does not assume that toxicants and nutrients affect the same molecular target, only that they affect the same epidemiologic outcomes. The Faroes study would seem to be particularly suited for such considerations because the correlation between fish intake and methylmercury exposure is relatively low, due to the fact that whale meat, rather than fish, is the main source of methylmercury exposure (Grandjean et al. 1992). Because fish intake and mercury exposure do not show a close correlation, separation of positive and negative effects on brain development would be possible by structural equation modeling. Still, although the effects of fish intake were in the direction predicted, statistically significant associations were observed only for spatial and motor function. However, the power to identify an effect of this confounder is limited by the imprecision of the crude questionnaire variable. This imprecision also causes an underestimation of the fish-adjusted mercury effect.

In the absence of detailed data on nutrient absorption levels, the validity of the questionnaire response on fish-dinner frequency cannot be determined. However, the imprecision is likely to be substantial. Previous studies of prenatal methylmercury exposure biomarkers have shown that imprecision varies from about 25% (cord blood) to about 50% (maternal hair) when expressed as the coefficient of variation (Budtz-Jørgensen et al. 2002; Grandjean et al. 2005a). The imprecision was even greater for the questionnaire response on whale-meat–dinner frequencies as a predictor of mercury exposure. The imprecision value for hair mercury corresponds to a reliability ratio of 0.68 (Budtz-Jørgensen et al. 2004). A greater imprecision (and lower reliability ratio) would seem plausible for the questionnaire response on fish dinners as a proxy for nutrient intakes. As illustrated by Equation 3, ignoring fish error in the standard analysis of outcomes with a strong fish effect will yield mercury effect estimates that underestimate the adverse effect.

In regard to motor function outcomes, inclusion of fish intake as a confounder with a realistic reliability ratio of 43% doubled the mercury effect compared with results unadjusted for fish intake. For the spatial function, which in the unadjusted analysis seemed to benefit slightly from mercury exposure, the adjustment resulted in a negative regression coefficient in accordance with the anticipation of mercury toxicity. Other outcomes were not significantly associated with fish intake, and the adjustment was therefore of less importance. Still, an attenuation of mercury effects on functional domains other than motor function would be plausible. For example, previous studies of fish intake have suggested beneficial effects mainly on visually mediated functions and, possibly, general intelligence (Daniels et al. 2004; Oken et al. 2005; Willatts and Forsyth 2000). However, because of the absence of better nutrient supply data, the present study cannot further elucidate this potential.

Two other long-term prospective studies of developmental methylmercury neurotoxicity have been carried out and have been used for risk assessment [National Research Council (NRC) 2000]. In New Zealand, groups of mothers with the same high fish intake were compared in regard to different levels of methylmercury exposures (Kjellström et al. 1989). Because this study incorporated matching for fish intake, it may be affected to a lesser degree by confounding from nutrient intakes. In the Seychelles, where the average fish intake is high and causes average methylmercury exposures higher than in the Faroes (Shamlaye et al. 1995), the confounding may be greater (Clarkson and Strain 2003). In this regard, the New Zealand results (Kjellström et al. 1989) suggested a stronger mercury effect than the Faroes study (before adjustment for fish intake), whereas the Seychelles study showed some associations that suggested a beneficial effect of mercury exposure (Davidson et al. 1998). However, comparisons between these studies must also take into account differences in exposure assessment, sources of bias, and the sensitivity of the outcome parameters to subclinical neurotoxicity (Grandjean et al. 2005b). Although the findings of these three studies are not necessarily conflicting (Keiding et al. 2003; NRC 2000), perhaps the results would be more similar if the (confounding) effects of nutrients could be separated from the effects of methylmercury.

The present analysis therefore emphasizes that the opposite effects of beneficial nutrients and toxic contaminants should not be ignored by epidemiologic studies in this field. Because of the positive correlation between beneficial and hazardous exposures, confounding will invariably occur. Depending on the correlation between exposure and confounder, and the impact of the confounder on the outcome parameters, underestimation of the effects of one factor will occur if adjustment for the other is not included. Furthermore, an unbiased assessment of the contaminant toxicity also requires that account is taken of the imprecision, both of the contaminant exposure and of the proxy variable that reflects the confounding factor.

The confounding issue has only recently surfaced in publications on the advantages of seafood diets and the risks of marine contaminants (European Food Safety Authority 2005). Also, the claim has been made that adverse effects of methylmercury on children’s neurobehavioral functions do not occur when the mercury originates from a diet based on ocean fish (Clarkson et al. 2003). The present study suggests that uncontrolled confounding, and imprecision of the confounder, will bias the mercury toxicity findings toward the null, and that the extent of this bias will be study dependent.

This issue may be relevant beyond childhood neurobehavioral development. Thus, in regard to cardiovascular health, a small number of epidemiologic studies have assessed both fatty acid intakes and mercury exposures. Thus, evidence on cardiovascular mortality suggests that fatty acids and methylmercury from fish act in different directions and that mercury exposure may cancel the benefits from a fish diet (Guallar et al. 2002; Virtanen et al. 2005). This issue was also examined in a U.S. cohort of health professionals, including dentists with occupational exposure to mercury vapor; support for a mercury effect was seen only in the nondentists (Yoshizawa et al. 2002). Because of misclassification of methylmercury exposure (e.g., based on mercury concentrations in toenails), these studies also most likely underestimate the true effect of methylmercury exposure.

We recommend that future studies assess both beneficial and detrimental effects of seafood intake at the same time, in an attempt to separate opposite impacts on the outcomes. Further, some cautious judgment is possible at this time. The analyses presented here suggest that previously published results from the Faroes prospective study (e.g., Grandjean et al. 1997) underestimate the true extent of developmental methylmercury neurotoxicity. Although previous reviews have emphasized the possibility of overestimation of toxicity (National Toxicology Program 1998; Smith and Sahyoun 2005), only minor bias in this direction has been subsequently identified (Budtz-Jørgensen et al. 2002, 2007; Grandjean et al. 2001). In contrast, because of the imprecision of the exposure parameters, the mercury effect is underestimated and the benchmark dose results are overestimated (Budtz-Jørgensen et al. 2004), thereby possibly resulting in exposure limits with less protection than intended. The present study shows that bias from lack of confounder adjustment for nutrient intakes further adds to the underestimation of methylmercury neurotoxicity.

A final issue deserves attention in regard to fish advisories and dietary recommendations. Although these statements may seem difficult to reconcile because of the presence of both mercury and beneficial nutrients in fish and seafood, both deserve attention, perhaps even more so, because the health impact of both is most likely underestimated. The consumer is therefore well advised to include seafood and freshwater fish in the diet in order to obtain the benefits, but to choose fish and seafood low in contaminants. Fortunately, fish with a high content of beneficial fatty acids do not necessarily contain much mercury (Gochfeld and Burger 2005; Levenson and Axelrad 2006; Mahaffey 2004; Smith and Sahyoun 2005), and a prudent choice is therefore possible, given appropriate guidance. In the Faroe Islands, the health authorities issued an advisory that recommends women in fertile age groups to abstain from eating whale meat, and this advisory has resulted in decreased exposure levels (Weihe et al. 2005).

## Figures and Tables

**Table 1:** Mercury effects on neurobehavioral tests at 7 and 14 years of age, as determined in structural equation analysis with covariate adjustment before and after addition of the frequency of maternal fish dinners during pregnancy.

			Mutual adjustment			
	Mercury without adjustment for fish intake		Fish intake		Mercury	
Age/test group	Effect^a^	*p*-Value	Effect	*p*-Value	Effect	*p*-Value
7 Years						
Motor	−9.74	0.034	25.1	0.010	−12.2	0.0092
Verbal	−10.4	0.0018	3.62	0.61	−10.8	0.0017
14 Years						
Motor	−7.41	0.033	19.9	0.006	−9.37	0.0082
Attention	−8.40	0.029	12.2	0.13	−9.54	0.016
Spatial	2.60	0.50	17.3	0.031	1.04	0.79
Verbal	−5.97	0.080	9.85	0.16	−6.87	0.049
Memory	−2.86	0.39	3.15	0.64	−3.05	0.37

a Effect of true exposure doubling expressed in percent of SD of latent response.

**Table 2:** Mercury effects on neurobehavioral tests at 7 and 14 years of age, as determined in structural equation analysis with covariate adjustment that includes maternal fish intake during pregnancy at different levels of precision (indicated by the reliability ratio).

	7 Years		14 Years			
	Motor function		Motor function		Spatial function	
Precision of fish nutrient intake (%)	Mercury effect^a^	*p*-Value	Mercury effect	*p-*Value	Mercury effect	*p-*Value
100	−12.2	0.0092	−9.37	0.0082	1.04	0.78
68	−13.7	0.0048	−10.7	0.0036	0.088	0.96
43	−17.0	0.0017	−13.6	0.0009	−1.57	0.73
27	−23.7	0.0006	−20.1	0.0003	−4.18	0.51

a Effect of true exposure doubling expressed in percent of SD of latent response.
